# Modeling Case Burden and Duration of Sudan Ebola Virus Disease Outbreak in Uganda, 2022

**DOI:** 10.3201/eid3109.241545

**Published:** 2025-09

**Authors:** Donal Bisanzio, Henry Kyobe Bosa, Barnabas Bakamutumaho, Carolyne Nasimiyu, Diana Atwine, Daniel Kyabayinze, Charles Olaro, Robert F. Breiman, M. Kariuki Njenga, Henry Mwebesa, Jane Ruth Aceng, Richard Reithinger

**Affiliations:** RTI International, Washington, DC, USA (D. Bisanzio, R. Reithinger); Ministry of Health, Kampala, Uganda (H.K. Bosa, B. Bakamutumaho, D. Atwine, D. Kyabayinze, C. Olaro, H. Mwebesa, J.R. Aceng); Washington State University, Pullman, Washington, USA (C. Nasimiyu, M.K. Njenga); Emory University, Atlanta, Georgia, USA (R.F. Breiman)

**Keywords:** Ebola virus, viruses, zoonoses, Sudan Ebola virus, Sudan virus, SUDV, *Orthoebolavirus sudanense*, hemorrhagic fever, modeling, Sudan, Uganda

## Abstract

In 2022, a Sudan Ebola virus outbreak was confirmed in Uganda. Within 1 month of the outbreak’s onset, we developed an individual-based modeling platform to estimate the unfolding outbreak’s burden of cases and deaths, as well as its duration, using different scenarios. Modeled projections were within the range of observed cases.

Ebola virus disease (EVD) is a severe, often fatal illness affecting humans and primates (*1*). In the past 4 decades, 36 EVD outbreaks have occurred across 11 countries, resulting in >15,000 deaths (*2*). With case-fatality rates that can reach >65%, EVD is among the most lethal viral hemorrhagic fevers.

On September 20, 2022, an outbreak of Sudan Ebola virus (SUDV; *Orthoebolavirus sudanense*) in southcentral Mubende District, Uganda, was confirmed by the Uganda Ministry of Health (MOH) (*3*,*4*); cases rapidly spread to 8 nearby districts. By mid-October, concerns within the MOH and the international community about the potential magnitude of the outbreak accelerated when a treatment-seeking infected person traveled to the highly populated capital city, Kampala; many new cases were linked to that patient (*5*), who eventually died. Because no effective treatment or vaccine existed against SUDV (*6*), the MOH’s response to mitigate the outbreak relied on nonpharmaceutical interventions (NPIs), including aggressive case isolation and contact tracing; safe burials; hygiene promotion; social and behavior change; and lockdowns. NPIs were applied on the basis of successful experiences from previous EVD outbreaks in sub-Saharan Africa and built upon the prevailing COVID-19 pandemic response infrastructure (*7*). The aim of this study was to develop—during the first month after the outbreak started—a methodological approach to rapidly predict the epidemic curve and burden of the SUDV outbreak, depending on the timing and intensity of the interventions by local health officials.

## The Study

We modified a well-characterized individual-based model (IBM) framework, previously used to estimate disease burden for COVID-19 (*8*), mpox (*9*), and Ebola (*10*), for the purposes of the SUDV outbreak (IBM-SUDV; Appendix). Unlike ordinal differential equation models or other IBMs previously published for Ebola (*11*), IBM-SUDV included the geographic distribution and movement of the Uganda population, as well as a contact network representing population interactions at local and regional levels, using available demographic data and accounting for heterogeneity in interactions among age groups. IBM-SUDV included uptake and impact of NPIs, simulating the response to the 2022 SUDV outbreak in Uganda, such as contact tracing, case isolation, safe burial, and use of personal protective equipment (PPE). We modeled timing of intervention deployment and response heterogeneity to estimate the outbreak’s case burden, deaths, and duration.

We modeled SUDV transmission using the classical susceptible→exposed→infectious→recovered compartmental model structure (*8*–*10*). The transition from one status to another was a function of pathogen characteristics (e.g., probability of effective transmission per close contact, incubation period, infectious period, and fatality rate) and interaction among persons (only for susceptible to infectious). For all persons, we determined parameter values for treatment-seeking behavior, hospitalization, fatality, and burial by using either published data or estimates. This model represented the baseline scenario. We then compared the baseline scenario to 2 hypothetical scenarios: a delayed outbreak response scenario, which assumed a 5-month delay in reaching the coverage and uptake of the 2022 SUDV outbreak NPI response; and an out-of-control outbreak scenario, which assumed a 5-month delay in having NPIs in place, as well as a mean 50% contact tracing and isolation rate (i.e., similar to what was observed in the early phase of the 2014–2016 West Africa Ebola outbreak).

For each scenario, we completed 1,000 simulations with a time horizon of 150 weeks each. For each scenario, we estimated the median number of cases, hospitalizations, and deaths, as well as the outbreak’s median duration. The outbreak’s duration was determined from the occurrence of the first case to the time at which zero cases were observed 42 days after the last infection event. For each outcome, we calculated a 95% credible interval (CrI) using the adjusted bootstrap percentile approach (*12*).

We compiled and visualized the predicted epidemic curve for each of the 3 scenarios (Table; Figure). With NPIs implemented, the baseline scenario estimated a median number of 193 (95% CrI 131–277) cases and 81 (95% CrI 55–124) deaths; the outbreak’s median duration was 22 (95% CrI 14–25) weeks (Figure, panel A). The delayed outbreak response scenario estimated 778 (95% CrI 665–901) cases and 303 (95% CrI 259–351) deaths, and the outbreak’s median duration extended to 24 (95% CrI 20–28) weeks (Figure, panel B). The out-of-control outbreak scenario estimated 13,537 (95% CrI 9,376–19,919) cases, 5,279 (95% CrI 3,656–7,768) deaths, and a median outbreak duration of 24 (95% CrI 22–27) months (Figure, panel C). The IBM-SUDV’s modeled projections were completed on November 1, 2022, and shared with the Uganda MOH on November 11, 2022.

**Table:** Comparison of predictions obtained from modeling case burden and duration of Sudan Ebola virus disease outbreak in Uganda, 2022*

Scenarios	No. cases (95% CrI)	No. deaths (95% CrI)	Epidemic duration (95% CrI)
2022 Ebola outbreak†	164	77	16.5 wk
Individual-based Uganda model‡			
Baseline	193 (131–277)	81 (55–124)	22 wk (14–25 wk)
Delayed response	778 (665–901)	303 (259–351)	24 wk (20–28 wk)
Out-of-control	13,537 (9,376–19,919)	5,279 (3,656–7,768)	24 mo (22–27 mo)

*CrI, credible interval. †Reported burden and duration. ‡Predicted burden and duration.

**Figure:**
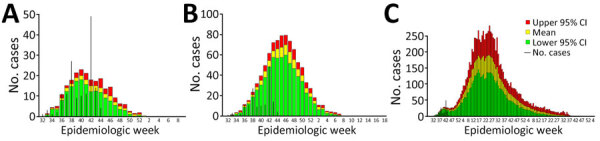
Predicted individual-based model epidemic curves compared with actual epidemic data for study modeling case burden and duration of Sudan Ebola virus (SUDV) disease outbreak in Uganda, 2022. A) Baseline scenario, simulating the actual response to the outbreak, including timing of nonpharmaceutical interventions (NPIs; i.e., contact tracing, isolation, personal protective equipment). B) Delayed outbreak response scenario, which assumed a 5-month delay in reaching the NPI coverage and uptake of the 2022 SUDV outbreak. C) Out-of-control scenario, which assumed a 5-month delay in having NPIs in place, as well as a mean 50% contact tracing and isolation rate. Black bars indicate actual numbers of cases reported during the outbreak.

Before 2022, Uganda had reported 6 EVD outbreaks (*2*). Of the reported outbreaks, 4 were caused by SUDV (2000, 2011, and 2012 [n = 2]), 1 was caused by Bundibugyo virus (*O. bundibugyoense*; 2007), and 1 was caused by Zaire Ebola virus (*O. zairense*; 2019). Those outbreaks’ median number of cases was 8.5 (range 1–425) and median deaths was 4 (range 1–224); in total, the outbreaks resulted in 596 cases and 273 deaths (*2*).

For the 2022 SUDV outbreak, the actions of the MOH swiftly halted virus circulation in all affected districts (*5*,*13*). Ebola treatment units (ETUs) were activated at Mubende and Fort Portal Regional Referral Hospitals on September 20, 2022, and symptomatic contacts were evacuated directly to ETUs for testing. Entebbe Regional Referral Hospital activated its ETU on October 6, 2022, and Mulago National Referral Hospital’s ETU was activated on October 15. Early in the outbreak, most cases were healthcare-associated rather than household-associated; cases resulting from burial or vertical or sexual transmission were rare (*13*). The 2022 SUDV outbreak lasted 69 days and caused 164 cases and 77 deaths (*3*). The mean age of case-patients was 28 years, and the highest case-fatality rates were observed among children <10 years of age (75%) and adults 40–49 years of age (61.5%). Uganda’s MOH officially declared the outbreak’s end on January 11, 2023, sixty days after the last infection event.

## Conclusions

During the 2022 SUDV outbreak in Uganda, within 1 month of the first case being confirmed, we developed the IBM-SUDV to model the burden and duration of the outbreak. The outbreak’s reported numbers of cases and deaths (164 cases and 77 death reported vs. 193 [95% CrI 131–277] cases and 81 [95% CrI 55–124] deaths modeled), as well as the average duration (16.5 weeks reported vs. 22 [95% CrI 14–25] weeks modeled), were within the ranges of our baseline scenario. Delayed outbreak and out-of-control outbreak scenarios would have resulted in a substantially greater outbreak burden and duration, similar to the 2014–2016 EVD outbreak across Guinea, Liberia, and Sierra Leone (i.e., 28,600 cases and 11,325 deaths). The model highlighted the importance of a rapid response to effectively control the outbreak, which ultimately occurred (*5*,*13*). After we shared model results with MOH in early November, the MOH intensified NPI implementation, particularly contact isolation and an unprecedented lockdown of 2 hotspot districts. Further IBM-SUDV modeling is being discussed with the MOH, including to determine SUDV transmission dynamics more accurately, using actual 2022 SUDV outbreak clinical, epidemiologic, and operational response data (e.g., from individual cases and contact tracing) (*4*,*5*,*14*), as well as estimate the effectiveness of changing various NPI parameters, additional NPIs, and available therapeutic options (e.g., vaccine).

In summary, our model estimated that the MOH’s prompt response to this outbreak averted up to 13,000 cases and 5,000 deaths. The effective response was likely aided by Uganda’s prior experience responding to EVD outbreaks (*3*), a national disaster preparedness and management policy (https://faolex.fao.org/docs/pdf/uga171437.pdf), a public health emergency operations center and relevant task forces (*15*), external partner coordination, and infrastructure and resources built during the COVID-19 pandemic (*7*).

Appendix Additional information for modeling case burden and duration of Sudan Ebola virus disease outbreak in Uganda, 2022.
