# Supplementary material for: Modeling Case Burden and Duration of Sudan Ebola Virus Disease Outbreak in Uganda, 2022
Source: Emerg Infect Dis. 2025 Sep;31(9):1829–32. doi: 10.3201/eid3109.241545 (PMC12407115; doi:10.3201/eid3109.241545)
Supplement: Appendix — Additional information for modeling case burden and duration of Sudan Ebola virus disease outbreak in Uganda, 2022. [file 24-1545-Techapp-s1.pdf]

Article DOI: https://doi.org/10.3201/eid3109.241545

EID cannot ensure accessibility for supplementary materials supplied by authors. Readers who have difficulty accessing supplementary content should contact the authors for assistance.

Modeling Case Burden and Duration of Sudan Ebola Virus Disease Outbreak in Uganda, 2022

Appendix

Additional Methods

1.1 Model Overview

An individual-based model (IBM) (RTI Cassandra^{TM}; https://github.com/dadedodo/Cassandra-RTI) was developed to (i) estimate the burden of cases and deaths, as well as (ii) the duration of the unfolding 2022 Sudan ebolavirus (SUDV) disease outbreak in Uganda (UGA). Additionally, we evaluated the possible effect of non-pharmaceutical interventions (NPIs) on outbreak dynamics. The IBM-SUDV structure was based on a contact network representing interactions of people at the local level (e.g., within households, schools, and local businesses) and at the regional level (e.g., movement between villages and cities). The resolution levels allow the model to account for contact heterogeneity among people while maintaining a manageable level of abstraction (and subsequently less computational complexity and faster run times).

There are three main reasons for choosing an IBM instead of, for example, an ordinary differential equations (ODE)-based model. First, IBMs can effectively describe the effect of super-spreaders in the transmission dynamics of contact-borne infectious diseases (*1*,*2*). The impact of super-spreaders is well-known in ebolavirus transmission, and failing to capture this characteristic will result in an underestimation of the effect of response interventions, including contact tracing (*3*). A main assumption in ODE models is that the simulated population is "well-mixed," meaning all individuals have the same number of contacts—a known incorrect assumption. Rather, in contact-borne infectious diseases, the number of contacts per individual follows a long-tailed distribution (i.e., following a power law or Pareto distribution) (*4*). This

heterogeneity in a population's contact distribution is due to some individuals having a high number of contacts and commonly being responsible for 80% of infection events (*5*,*6*). Second, IBMs offer flexibility in describing the characteristics of the simulated population, their short- and long-range movement, and varying intervention efforts across locations (*7*). As described below, our model includes three main subpopulations (general population, healthcare workers, and frontline workers), various locations (households, health centers, schools), and different interventions with their implementation times. It would have been extremely difficult to create such a model using an ODE structure due to the number of equations required to represent all these characteristics, movements, and interventions. Third, an advantage of selecting an IBM is the simplicity of explaining its structure to those without advanced modeling training. IBMs are rule-based models that can be easily described without complex formulas. This is beneficial when epidemiologists and modelers collaborate with public health practitioners and policy makers who lack modeling experience. We have successfully applied the same IBM framework to model the transmission dynamics of Ebola in DRC (*8*), COVID-19 in Saudi Arabia (*9*), and other contact-borne infectious diseases in different settings, closely collaborating and engaging with countries' Ministries of Health to ensure that the developed models reflected available disease-specific and operational data, as well as country priorities and interests.

The IBM-SUDV contact network includes one node per person in the Ugandan population, estimated to be 47.3 million people (*10*). The links between nodes represent possible contacts between people and were created using publicly available and free geodata obtained by remote sensing and scientific literature. Geodata used by the model included population density maps produced by WorldPop (*11*) and Facebook (*12*) as well as accessibility maps produced by the Malaria Atlas Project (MAP) (*13*).

The following provides a brief overview of the steps used to create the IBM:

▪ Identified human settlements (cities and villages), including their geographic size (km^2) and population, using density maps obtained from WorldPop (*11*). To improve the model run time, the country was then divided into 10 km x 10 km tiles. The tiles represented the "settlement" units in the IBM. Villages typically fit within one tile, while a city may span many tiles. The density was adjusted to reflect the estimated population in 2022.

▪ Created a dynamic network for each human settlement tile representing transmissible contacts of people on a per-week basis (intra-settlement network) and parameterized the network using information published in the scientific literature; this network represented short-range movement (Appendix Figure). The IBM accounted for changes in contact during an outbreak and included preintervention and postintervention periods to account for interventions adopted during an outbreak to reduce the spread of the disease.

▪ Created links among people living in different human settlement tiles (extra-settlement network) using data from MAP's accessibility map and WorldPop's migration map and further merged with information from scientific literature; the links created in this process represent in-country, long-distance movement.

The human social network and the data used to characterize the network are described in more detail in Section 1.2 and Section 1.3.

SUDV transmission in the IBM was modeled using the classical SEIR compartmental model structure: Susceptible (*S*) → Exposed (*E*) → Infectious (*I*) → Recovered (*R*). Infectious individuals were at risk of dying, and individuals who died remained infectious until they were buried. The transition from one status to another was a function of pathogen characteristics (e.g., probability of effective transmission per close contact, incubation period, infectious period, and fatality rate [see Appendix Table 3]) and interaction among individuals (only for *S* to *I*).

The following steps were taken to allow the IBM to account for SUDV transmission:

▪ Estimated the number of treatment units (clinics and hospitals, not specific to SUDV) and healthcare workers (HCW) per human settlement tile, based on publicly available data from the World Health Organization (WHO) and published literature, to allow the IBM to represent human treatment-seeking behavior in the case of symptomatic Ebola virus disease (EVD).

▪ Introduced the transmission of SUDV in the IBM using parameters from published literature.

▪ Introduced the modification of human behavior (i.e., a dynamic social network) linked to SUDV infection.

Once the IBM was programmed with SUDV transmission and adopted interventions, the model was calibrated comparing outcomes with available reported data. Calibration was

performed using trend analysis comparing the reported data and the results obtained from the IBM-SUDV. The trend of obtained and simulated data was estimated using a generalized additive model (GAM). The trend analyses were performed to estimate the smooth trend of Ebola cases across the first 13 weeks of the outbreak. The model was built using case counts as independent variable, and epidemic week as nonlinear dependent variable. The result of the GAM was the mean expected number of cases per epidemiologic week and 95% confidence interval. We performed two GAM, one using reported cases and one using data obtained from 1,000 IBM-SUDV simulations. The mean number of cases per epidemiologic week obtained from GAM for reported and simulated cases were compared using Kendall's concordance test. We considered the model well-calibrated when Kendall's W reached a value above 0.9.

Free, open-source software was used to program the IBM. The geodata was analyzed and stored using the free, open-source Geographic Resources Analysis Support System (GRASS) software (https://grass.osgeo.org). Data analysis was programmed using the R programming language (The R project for Statistical Computing, https://www.r-project.org). The IBM structure was programmed using the Julia programming language. Julia is a programming language suitable for IBMs because of its high speed, easy-to-use packages, and clear writing style (J. Bezanson et al., unpub. data, https://arxiv.org/abs/1209.5145). The IBM-SUDV was run on a high-performance computer.

1.2 Human Social Network Data

1.2.1 Population Data

Data on the Ugandan population were obtained from World Bank (*10*) and disaggregated at into 10 km x 10 km tiles using data obtained from WorldPop (https://www.worldpop.org/). WorldPop provided population estimates broken down by gender and age groupings (including 0–1 years and by 5-year ranges up to 80+ years) at a resolution of 100 m. The estimates per each tile were adjusted to represent Uganda's estimated population of 2022. The data obtained from WorldPop were used to estimate the size of the network for each human settlement.

1.2.2 Human Settlements

To identify human settlements at high resolution, a population density map created by Facebook (*12*) was used. This map identified areas with buildings at a resolution of 30 m, as updated to 2019. Facebook population density map data were analyzed using raster analyses

tools of GRASS software (i.e., to identify the position of human settlements based on presence of buildings). To improve the model run time, the country was then divided into 10 km x 10 km tiles. The population in each tile was calculated by aggregating values from the density maps. The tiles represented the "settlement" units in the IBM—villages typically fit within one tile, while a city may span many tiles. A connectivity matrix among the tiles was created to represent the extra-settlement movement of individuals in Uganda.

1.2.3 Travel Time Between Settlements

The movement of people between human settlement tiles was based on population size and travel times. Population sizes were calculated using WorldPop and Facebook data, as described previously. Travel times were calculated using the friction surface map created by MAP (*11)*. The friction surface map contains the time that a person spends passing through a map pixel based on estimated land-based travel speeds at a resolution of 1 km. Using the friction surface map estimation, we calculated the travel time between tiles.

1.2.4 Demographic Data

The model accounted for several demographic characteristics that well represent the Ugandan population. Data on household size, percentage of children enrolled in schools, and the unemployment rate were obtained from the Global Data Lab (*14*), the United Nations Children's Fund (UNICEF) (*15*) and the World Bank (*10*). In the IBM-SUDV, age and gender of individuals were used to create its contact network and assign them to each population type, as described below. Additional population characteristics needed to define vaccination scenarios for preventive vaccination, such as the number of HCW, security forces personnel, and transportation workers, were also obtained from World Bank and WHO sources, as described in ***Section 1.3.2***.

1.3 IBM Population Network Structure

The IBM uses a dynamic human social network to describe interaction among individuals. The structure of the network is initially fixed, with all links between individuals assigned before the simulations begin. During each simulation run, the model becomes dynamic as EVD cases cause links to be "activated." For example, infected individuals can acquire links as they travel to find hospitals, interact with HCWs, or interact with frontline workers (FWs)

outside their fixed network. Thus, all simulations start with same initial network, but the network evolves differently during each simulated epidemic. The use of a fixed initial network reduces result noise and reflects the normal routine of a country during non- outbreak periods.

Given the SUDV transmission dynamics and the time lag of the surveillance to report cases, the model ran on a weekly time step. Weekly estimates also made it easier to compare IBM estimates with real data. The following sections provide details about the IBM human social network and its components.

1.3.1 Representing the Human Social Network (Intra-settlement Network)

The IBM captured the high heterogeneity of the contact network among people, which is the key driver of disease spread in communities. The IBM network was built using the "scale-free" and "small-world" characteristics described for several social networks. A scale-free network has a high fraction of nodes (representing individuals) connected to a low number of other nodes and a few nodes connected to high number of other nodes. The nodes that have high connectivity are so-called "super spreaders." The link distribution among nodes in the scale-free network followed a power-law distribution:

$$p(x) = x^{-\alpha}$$

where x is the number of links of a node, $p(x)$ is the probability distribution, and the exponent α is the scaling factor. For human social networks, the power-law distributions have exponent α values ranging from 2 to 3 (*16*). A network has a "small- world" characteristic when two nodes in the network can reach each other through a short sequence of connected nodes (called a "short path") (*17*).

Interactions among individuals occurred in specific locations, which may have a key role in the spread of disease agents. The locations in which people spend most of their time daily are households, workplaces, and schools (*18*,*19*).

However, interactions outside the routine locations (e.g., markets, restaurants, and theaters) are at the base of the small-world characteristic of human social networks. Age is another important factor that shapes the social network of an individual. People tend to have more interaction with individuals of the same age that they meet at school, workplaces, or in recreational locations (*20*) Thus, to be sure that the IBM accurately described the interaction

among people, it included the distribution of interaction among individuals, interaction at locations, and the effect of individuals' ages.

After the population size and distribution among age groups within a settlement tile were estimated, a contact network was created using the following steps:

▪ Households were created using the mean size of the family cluster. The number of people living together in each house was determined using a Poisson distribution with the mean equal to the mean number of household inhabitants in Uganda by sub-regions as reported in the Uganda National Household Survey 2019/2020 (national mean household size was equal to 4.6 inhabitants) (*21*).

▪ The number of links (i.e., contacts) for each individual was calculated based on a power-law distribution with α = 2.5. Given the unavailability of data on number of contacts per people in Uganda, we set '*a'* equal to 2.5, a value that it usually used to create simulation representing the heterogeneity of people interaction in a social networks (*16*).Because the formulation of a power-law distribution requires indicating a minimum value for *x*, the minimum number of contacts for each individual was set equal to the mean household size in Uganda.

▪ Once the number of links was determined for each individual, the distribution of these links to other individuals followed age-stratified contact matrices reported for Uganda in Prem et al. 2021 (*20*).

▪ A location attribute was assigned to each link based on the settlement tile in which the contact occurred. The IBM also assigned one of four location types to each link: household, workplace, school, or other. The probability of assigning a link to a particular group was based on demographic characteristics of the Ugandan population.

▪ Links defined by the power-law distribution represent contacts based on proximity rather than on close contact (e.g., touching). SUDV transmits through close contact, so the proportion of close contacts among all contacts (0.09) was estimated by analyzing the data provided in by Olu et al., 2016 and Wolfe et al., 2017 (*22*,*23*). The proportion of close contacts was estimated by dividing the total number of contacts by the number of reported close contacts.

1.3.2 Representing the High-Risk Population

Within each settlement tile, the model accounted for the fraction of the population that are at high risk of infection during Ebola outbreaks due to their job activities. The IBM high-risk population included HCWs (e.g., doctors, nurses, midwives, dentists, and pharmacists) and non-health FWs (e.g., armed forces, teachers, and transportation workers) (*24*). Only individuals with an age above 17 years were assigned to the high-risk population. The probability of infection for this population was affected by the probability of being in close contact with an infectious individual. As infectious individuals seek treatment in hospitals or other health facilities, the IBM's treatment-seeking module creates links to connect infectious individuals with HCWs and FWs. These created links establish the exposure to the virus for the high-risk population. Linear regression was used to estimate the trend in the number of HCWs per profession to estimate figures for 2022, when not available.

The number of non-health FWs was estimated by type (i.e., armed forces, transportation workers, and other FWs) from published and public sources where possible (Appendix Table 1). No data were available for the number of other FWs, such as shop and market workers, clergy, contact tracers, and burial teams, so the model assumed a density of 3.5 per 1,000, which is between the density of HCWs (4.2 per 1,000) and transportation workers (2.5 per 1,000). The model included parameters capturing interaction in schools and universities. We calculated the number of teachers and professors and their interaction with students using data of the Ugandan education system. We gathered data on the ratio of number of students per teachers / professor and school / university enrollment (*10*). Next, to reflect that non-health FWs typically have many contacts in the community, these workers were assigned in the network from among individuals with the greatest number of contacts. Specifically, the IBM randomly selected individuals to be FWs from among those individuals in the 80th percentile for number of contacts. This selection was completed for each tile.

Appendix Table 1 reports the number of individuals in each high-risk population group.

1.3.3 Representing Human Movement Among Settlements

The extra-settlement spread of SUDV in the IBM was captured using a weighted network that links settlement tiles (Appendix Figure). The weight of each link was determined by the estimated flux of people between tiles. Data about individual movement within Uganda were not

available, so the IBM applied a gravity model that accounted for distance between pairs of settlements, travel times, and population sizes (*25–27*). A gravity model is a modified law of gravitation that, in a simpler formulation (frictionless gravity model), considers the population size of two places and their distance apart to estimate the flow of people between them (*28*). This approach is not country specific but has been used in the past in West Africa (*27*). Larger settlements were assumed to attract more people than smaller settlements, and settlements closer together were assumed to share more people than settlements further apart. However, in settings where connections among places are not easy, the model can be adjusted by adding travel times (friction-based gravity model) (*28*).

The distance and travel time between each pair of settlement tiles was calculated using the population density map (*12*) and the surface friction map (*13*). The weight of each link was used to calculate the probability that Ebola cases will move between two settlement tiles, causing the outbreak to spread to new locations. The extra-settlement network was created using the following steps:

▪ Created a distance matrix among settlement tiles with columns and rows equal to the number of settlement tiles. Each matrix cell contained the distance between two settlement tiles.

▪ Created a travel time matrix among settlement tiles with columns and rows equal to the number of settlement tiles. Each matrix cell contained the travel time between two settlement tiles.

▪ Built a gravity model merging population data, the distance matrix, and the travel time matrix following the methods described in Balcan et al. (*26*) and Kraemer et al. (*27*).

▪ Recorded the results of the gravity model in a flux matrix with columns and rows equal to the number of settlement tiles. Each matrix cell contained the estimated flow between two settlement tiles. This matrix is not symmetric because the model uses tile population as an attraction factor. Thus, tiles with high population density have a greater inward flow than tiles with low population density.

Appendix Table 2 summarizes the parameters and data sources for the IBM intra-settlement and extra-settlement networks.

1.4 Modeling SUDV Transmission

1.4.1 Background

SUDV transmission depends on disease-specific characteristics, such as transmission probability per close contact, incubation period, and infectious period. Transmission is also affected by factors linked to population demographics and behavior (e.g., characteristics of the social network, treatment-seeking behavior, and burial practices), individual mobility, and healthcare system capacity. The IBM accounted for these factors to modulate the dynamic transmission of Ebola and affect the size of outbreaks. The model also included preintervention and postintervention periods to account for interventions adopted during an outbreak to reduce the spread of the disease, including ring vaccination, safe burials, case isolation, contact tracing, extensive testing, HCW training, and availability of personal protection equipment (PPE). ***Section 1.4.2*** describes the modeling of SUDV transmission, and ***Section 1.4.3*** provides details about SUDV interventions included in the model.

1.4.2 SUDV Transmission

In the model, each simulation starts with one infected individual selected at random. SUDV then transmits within the population as individuals interact. Because close contacts between individuals may vary in duration and proximity, only a fraction result in transmission. Studies have estimated that Ebola transmission probability could range from 0.45 to 0.55 (*29*,*30*). Thus, given the transmission uncertainty, the probability of effective transmission per close contact was randomly selected for each run of the IBM-SUDV between 0.45 and 0.55 using a uniform distribution (Appendix Table 3). Once infected, individuals with new cases of EVD may experience hospitalization or death.

Some individuals have infection-conferred immunity after surviving an Ebola infection during a previous outbreak. Since 2000, a total of 596 cases and 273 deaths have been reported in Uganda (*31*). Assuming 20% of cases were asymptomatic, estimated from Richardson et al., 2016; Mbala et al., 2017 (*32*,*33*), the number of recovered cases since 2000 is 442. Accounting for deaths, an estimated 220 individuals are currently living with infection-conferred SUDV immunity in Uganda (*31*) (Appendix Table 3). Given the low number of publications describing transmission dynamics of SUDV, the parameters used in the IBM-SUDV were estimated using

articles describing epidemics caused by the Zaire or the Sudan virus. A summary of the parameters and data sources for Ebola transmission is provided in Appendix Table 3.

1.4.3 SUDV Interventions

The model included NPIs as implemented during the last SUDV outbreaks in Uganda; a therapy or vaccine against SUDV disease was not available in 2022. Interventions from the 2018–2020 outbreaks in the DRC were chosen as it was assumed that similar interventions would be used in Uganda, in accordance with current guidelines (*41–43*). Parameters for all interventions are reported in Appendix Table 4.

NPI included the use of PPE for HCWs, contact tracing of infected individuals, safe burials, and increased treatment seeking due to increased awareness in the population (Appendix Table 4). These interventions were simulated using information about the strategies employed during the last EVD outbreaks in Uganda. To capture this heterogeneity, each settlement tile was randomly assigned a percentage between 20% and 70% (uniform distribution) of contacts of infected individuals successfully located by contact tracing teams (estimated from WHO, 2019b, and WHO, 2020) (*42*,*43*). In the IBM, when a new case of EVD was identified within a settlement tile, linked contacts of the infected individual were randomly selected using this contact-traced percentage and designated as the contact-traced group. Individuals in this group represent those who were monitored for 21 days and hospitalized if showing any symptoms. Thus, individuals in the contact-traced group were not able to infect others.

Overall, NPIs were started in the model 1 week after the first reported case. Full implementation of NPIs in the model was reached within 40 weeks.

1.5 IBM Assumptions

The model included several assumptions to populate variables for which data were not available, including the following:

▪ As it is not possible to define urban and rural areas explicitly, settlements with over 10,000 inhabitants were assumed to be urban areas.

▪ At least one healthcare worker was present in each settlement.

▪ Hospitals and clinics with sufficient capacity to handle Ebola cases were located only in urban settlements. Cases requiring hospitalization were in contact with healthcare workers in the closest urban settlement.

▪ Because the size of hospital crews var.y, the model assumed the size of hospital crews followed a truncated Poisson distribution, with at least 2 people and a mean of 3 people (information obtained by personal communication with Médecins Sans Frontières personnel).

▪ Armed forces were present in urban settlements, and personnel were assigned proportionally to the settlement's population size.

▪ The number of people who had contact with the body during a funeral followed a Poisson distribution with a mean of 10. This assumption was relevant only for traditional burials, as the model assumed transmission did not occur during safe burials.

1.6 Model Outcomes

The results of each scenario tested with the IBM-SUDV were based on 1,000 runs. We tested the number of runs to obtain robust results. The evaluation of association between model runs and result robustness by comparing the uncertainty of a dummy scenario obtained from 50 to 1,000 runs. The results showed that the uncertainty of model results did not sensibly reduce after 1,000 runs (Appendix Figure).

The IBM estimated the median number of symptomatic cases and deaths as well as the median duration of the epidemic for each scenario analyzed. The epidemic duration was measured from the first case to the time at which zero cases remained.

Additionally, a 95% credible interval (CrI) was calculated for each outcome. CrIs are estimated in situations where the parameter is a random variable. CrIs then represent the interval within which an unobserved parameter value falls with a given probability. In comparison, confidence intervals are estimated in situations with known, observed data, where intervals can be precisely calculated. For the IBM, 95% CrIs were calculated using the adjusted bootstrap percentile approach (*48*) based on 10,000 re-samplings of the simulation results. This bootstrapping allows better estimates of the 95% CrIs because it calculates the CrIs from an estimated hypothetical distribution of the results.

Finally, the reduction in each outcome (and the 95% CrI of the reduction) was calculated for the comparison between several of the scenarios. The reductions and their 95% CrIs were calculated using the bootstrapping method described above (*48*).

Because of this, the reported reductions are not equivalent to the corresponding reductions in the median outcomes.

The Julia code used to build the IBM-SUDV can be provided upon request to Dr. Donal Bisanzio (dbisanzio@rti.org) and Dr. Richard Reithinger (reithinger@rti.org).

References

1. Chin WCB, Bouffanais R. Spatial super-spreaders and super-susceptibles in human movement networks. Sci Rep. 2020;10:18642. PubMed https://doi.org/10.1038/s41598-020-75697-z

2. Kim Y, Ryu H, Lee S. Agent-based modeling for super-spreading events: A case study of MERS-CoV transmission dynamics in the Republic of Korea. Int J Environ Res Public Health. 2018;15:2369. PubMed https://doi.org/10.3390/ijerph15112369

3. Lau MS, Dalziel BD, Funk S, McClelland A, Tiffany A, Riley S, et al. Spatial and temporal dynamics of superspreading events in the 2014–2015 West Africa Ebola epidemic. Proc Natl Acad Sci U S A. 2017;114:2337–42. PubMed https://doi.org/10.1073/pnas.1614595114

4. Großmann G, Backenköhler M, Wolf V. Heterogeneity matters: Contact structure and individual variation shape epidemic dynamics. PLoS One. 2021;16:e0250050. PubMed https://doi.org/10.1371/journal.pone.0250050

5. Woolhouse M. Quantifying transmission. Microbiol Spectr. 2017;5:5.4.07. PubMed https://doi.org/10.1128/microbiolspec.MTBP-0005-2016

6. Lloyd-Smith JO, Schreiber SJ, Kopp PE, Getz WM. Superspreading and the effect of individual variation on disease emergence. Nature. 2005;438:355–9. PubMed https://doi.org/10.1038/nature04153

7. Frias-Martinez E, Williamson G, Frias-Martinez V. An Agent-Based Model of Epidemic Spread Using Human Mobility and Social Network Information. Presented at: 2011 IEEE Third International Conference on Privacy, Security, Risk and Trust and 2011 IEEE Third International Conference on Social Computing; Boston, MA, USA; 2011 Oct 9–11.

8. Bisanzio D, Davis AE, Talbird SE, Van Effelterre T, Metz L, Gaudig M, et al. Targeted preventive vaccination campaigns to reduce Ebola outbreaks: An individual-based modeling study. Vaccine. 2023;41:684–93. PubMed https://doi.org/10.1016/j.vaccine.2022.11.036

9. Bisanzio D, Reithinger R, Alqunaibet A, Almudarra S, Alsukait RF, Dong D, et al. Estimating the effect of non-pharmaceutical interventions to mitigate COVID-19 spread in Saudi Arabia. BMC Med. 2022;20:51. PubMed https://doi.org/10.1186/s12916-022-02232-4

10. World Bank. 2022 population data [cited 2024 Oct 11]. https://data.worldbank.org

11. Lloyd CT, Chamberlain H, Kerr D, Yetman G, Pistolesi L, Stevens FR, et al. Global spatio-temporally harmonised datasets for producing high-resolution gridded population distribution datasets. Big Earth Data. 2019;3:108–39. PubMed https://doi.org/10.1080/20964471.2019.1625151

12. Facebook Connectivity Lab and Center for International Earth Science Information Network (CIESIN) - Columbia University (2022). Uganda: high resolution population density maps + demographic estimates [cited 2024 Oct 11]. https://data.humdata.org/dataset/highresolutionpopulationdensitymaps-cod

13. Weiss DJ, Nelson A, Gibson HS, Temperley W, Peedell S, Lieber A, et al. A global map of travel time to cities to assess inequalities in accessibility in 2015. Nature. 2018;553:333–6. PubMed https://doi.org/10.1038/nature25181

14. Global Data Lab. GDL Area Database 2016 [cited 2024 Oct 6]. https://globaldatalab.org/areadata/hhsize/COD/?levels=1%2B2%2B3%2B5%2B4&interpolation=1&extrapolation=1&extrapolation_years=3&nearest_real=0

15. United Nations Children's Fund (UNICEF). Country profiles: Uganda. 2022 [cited 2024 Oct 11]. https://data.unicef.org/country/cod

16. Barrat A, Barthélemy M, Vespignani A. Dynamical processes on complex networks, 1st edition. Cambridge: Cambridge University Press; 2008.

17. Kleinberg JM. Small-world phenomena and the dynamics of information. Adv Neural Inf Process Syst. 2002;14:431–8. https://doi.org/10.7551/mitpress/1120.003.0060

18. González MC, Hidalgo CA, Barabási A-L. Understanding individual human mobility patterns. Nature. 2008;453:779–82. PubMed https://doi.org/10.1038/nature06958

19. Wang Y, Yuan NJ, Lian D, Xu L, Xie X, Chen E, et al. Regularity and conformity: location prediction using heterogeneous mobility data. Presented at: 21st ACM SIGKDD International Conference on Knowledge Discovery and Data Mining; Sydney, NSW, Australia; August 10–13, 2015.

20. Prem K, Zandvoort KV, Klepac P, Eggo RM, Davies NG, Cook AR, et al.; Centre for the Mathematical Modelling of Infectious Diseases COVID-19 Working Group. Projecting contact matrices in 177 geographical regions: An update and comparison with empirical data for the COVID-19 era. PLOS Comput Biol. 2021;17:e1009098. PubMed https://doi.org/10.1371/journal.pcbi.1009098

21. Uganda Bureau of Statistics (UBOS). Uganda National Household Survey 2019/2020. Kampala: UBOS; 2021.

22. Olu OO, Lamunu M, Nanyunja M, Dafae F, Samba T, Sempiira N, et al. Contact tracing during an outbreak of Ebola virus disease in the Western area districts of Sierra Leone: lessons for future Ebola outbreak response. Front Public Health. 2016;4:130. PubMed https://doi.org/10.3389/fpubh.2016.00130

23. Wolfe CM, Hamblion EL, Schulte J, Williams P, Koryon A, Enders J, et al. Ebola virus disease contact tracing activities, lessons learned and best practices during the Duport Road outbreak in Monrovia, Liberia, November 2015. PLoS Negl Trop Dis. 2017;11:e0005597. PubMed https://doi.org/10.1371/journal.pntd.0005597

24. World Health Organization. Health workforce 2019 [cited 2020 Aug 1]. https://www.who.int/health-topics/health-workforce

25. Müller C, Doevenspeck M. The fast and the victorious: mobility, motorcyclists and political mobilisation in Uganda. Area. 2023;55:399–406. https://doi.org/10.1111/area.12872

26. Balcan D, Colizza V, Gonçalves B, Hu H, Ramasco JJ, Vespignani A. Multiscale mobility networks and the spatial spreading of infectious diseases. Proc Natl Acad Sci U S A. 2009;106:21484–9. PubMed https://doi.org/10.1073/pnas.0906910106

27. Kraemer MUG, Golding N, Bisanzio D, Bhatt S, Pigott DM, Ray SE, et al. Utilizing general human movement models to predict the spread of emerging infectious diseases in resource poor settings. Sci Rep. 2019;9:5151. PubMed https://doi.org/10.1038/s41598-019-41192-3

28. Anderson JE. The gravity model. Annu Rev Econ. 2011;3:133–60. https://doi.org/10.1146/annurev-economics-111809-125114

29. Xia ZQ, Wang SF, Li SL, Huang LY, Zhang WY, Sun GQ, et al. Modeling the transmission dynamics of Ebola virus disease in Liberia. Sci Rep. 2015;5:13857. PubMed https://doi.org/10.1038/srep13857

30. Rivers CM, Lofgren ET, Marathe M, Eubank S, Lewis BL. Modeling the impact of interventions on an epidemic of Ebola in Sierra Leone and Liberia. PLoS Curr. 2014;6: ecurrents.outbreaks.4d41fe5d6c05e9df30ddce33c66d084c.

31. Centers for Disease Control and Prevention. Ebola outbreak history [cited 2022 Oct 20]. https://www.cdc.gov/vhf/ebola/history/chronology.html

32. Richardson ET, Kelly JD, Barrie MB, Mesman AW, Karku S, Quiwa K, et al. Minimally symptomatic infection in an Ebola 'hotspot': a cross-sectional serosurvey. PLoS Negl Trop Dis. 2016;10:e0005087. PubMed https://doi.org/10.1371/journal.pntd.0005087

33. Mbala P, Baguelin M, Ngay I, Rosello A, Mulembakani P, Demiris N, et al. Evaluating the frequency of asymptomatic Ebola virus infection. Philos Trans R Soc Lond B Biol Sci. 2017;372:20160303. PubMed https://doi.org/10.1098/rstb.2016.0303

34. Eichner M, Dowell SF, Firese N. Incubation period of Ebola hemorrhagic virus subtype Zaire. Osong Public Health Res Perspect. 2011;2:3–7. PubMed https://doi.org/10.1016/j.phrp.2011.04.001

35. Malvy D, McElroy AK, de Clerck H, Günther S, van Griensven J. Ebola virus disease. Lancet. 2019;393:936–48. PubMed https://doi.org/10.1016/S0140-6736(18)33132-5

36. Conrad JR, Xue L, Dewar J, Hyman JH. Modeling the impact of behavior change on the spread of Ebola. In: Mathematical and statistical modeling for emerging and re-emerging infectious diseases. In: Chowell G, Hyman JH, editors. Mathematical and statistical modeling for emerging and re-emerging infectious diseases. Cham (Switzerland): Springer; 2016.

37. Legrand J, Grais RF, Boelle PY, Valleron AJ, Flahault A. Understanding the dynamics of Ebola epidemics. Epidemiol Infect. 2007;135:610–21. PubMed https://doi.org/10.1017/S0950268806007217

38. Agua-Agum J, Ariyarajah A, Blake IM, Cori A, Donnelly CA, Dorigatti I, et al.; WHO Ebola Response Team. Ebola virus disease among children in West Africa. N Engl J Med. 2015;372:1274–7. PubMed https://doi.org/10.1056/NEJMc1415318

39. Robert A, Camacho A, Edmunds WJ, Baguelin M, Muyembe Tamfum JJ, Rosello A, et al. Control of Ebola virus disease outbreaks: comparison of health care worker-targeted and community

vaccination strategies. Epidemics. 2019;27:106–14. PubMed https://doi.org/10.1016/j.epidem.2019.03.001

40. Potluri R, Kumar A, Maheshwari V, Smith C, Oriol Mathieu V, Luhn K, et al. Impact of prophylactic vaccination strategies on Ebola virus transmission: a modeling analysis. PLoS One. 2020;15:e0230406. PubMed https://doi.org/10.1371/journal.pone.0230406

41. World Health Organization. Meeting of the Strategic Advisory Group of Experts on Immunization, 22–24 March 2021: conclusions and recommendations. Wkly Epidemiol Rec. 2021;96:197–216.

42. World Health Organization. Strategic response plan for the Ebola virus disease outbreak in the provinces of north Kivu and Ituri. Geneva: WHO Press; 2019.

43. World Health Organization. Ebola virus disease—Democratic Republic of the Congo. Disease outbreak news: update. January 30, 2020 [cited 2020 Jul 2]. https://www.who.int/csr/don/30-january-2020-ebola-drc/en

44. Dunn AC, Walker TA, Redd J, Sugerman D, McFadden J, Singh T, et al. Nosocomial transmission of Ebola virus disease on pediatric and maternity wards: Bombali and Tonkolili, Sierra Leone, 2014. Am J Infect Control. 2016;44:269–72. PubMed https://doi.org/10.1016/j.ajic.2015.09.016

45. Jefferson T, Foxlee R, Del Mar C, Dooley L, Ferroni E, Hewak B, et al. Physical interventions to interrupt or reduce the spread of respiratory viruses: systematic review. BMJ. 2008;336:77–80. PubMed https://doi.org/10.1136/bmj.39393.510347.BE

46. Jalloh MF, Sengeh P, Bunnell RE, Jalloh MB, Monasch R, Li W, et al. Evidence of behaviour change during an Ebola virus disease outbreak, Sierra Leone. Bull World Health Organ. 2020;98:330–340B. PubMed https://doi.org/10.2471/BLT.19.245803

47. Chowell G, Nishiura H. Characterizing the transmission dynamics and control of Ebola virus disease. PLoS Biol. 2015;13:e1002057. PubMed https://doi.org/10.1371/journal.pbio.1002057

48. Davison AC, Hinkley DV. Bootstrap methods and their application. Technometrics. 1997;94:445.

Appendix Table 1. Uganda High-risk Population Estimated for the Year 2022*

Population	High-risk population per 1,000 population in Uganda (number of people)	Reference
HCWs		
Physicians	0.2 (9,460)	Estimated from WHO (*24*)
Nurses and midwives	0.5 (23,644)	Estimated from WHO (*24*)
Other healthcare professionals (e.g., dentists, pharmacists, lab workers, and physiotherapists)	0.6 (28,380)	Estimated from WHO (*24*)
Community HCWs	3.7 (175,010)	Estimated from WHO (*24*)
Total HCWs	4.9 (236,494)	Calculated
FWs		
Armed forces	0.9 (46,000)	World Bank (*10*)
Transportation workers	15.1 (709,500)	Müller & Doevenspeck (*25*)
Other FWs (e.g., shop and market workers, clergy, contact tracers, burial teams, teachers/professors)	3.5 (165,550)	Assumption
Total FWs	19.5 (921,050)	Calculated
Total high-risk population	24.4 (1,157,544)	Calculated

*FWs, frontline workers; HCWs, healthcare workers; UNICEF, United Nations Children's Fund; WHO, World Health Organization.

Appendix Table 2. IBM Population Network Parameters and Data Sources*

Model input	Input	Reference
Population	Estimated from the WorldPop (2020)	Lloyd et al., 2019 (*11*)
Demographic data	World Bank Open Data, UNICEF (2019)	UNICEF, 2022 (*15*)
Intra-settlement network		
Number of contacts per individual	Estimated using a power-law distribution with α = 2.5	Barrat et al., 2008 (*16*)
Contact matrix	Age-stratified, estimated	Prem et al., 2021 (*20*)
Probability that a contact is a close contact	0.09	Estimated from Olu et al., 2016; Wolfe et al., 2017 (*22*,*23*)
Extra-settlement network		
Number of settlements in Uganda	Estimated from the Facebook density map	Facebook CIESIN, 2019 (*12*)
Distance between settlements	Estimated from the Facebook density map	Facebook CIESIN, 2019 (*12*)
Travel time between settlements	Estimated using MAP friction map	Weiss et al., 2018 (*13*)
Extra-settlement flow of people	Estimated using the gravity model	Balcan et al., 2009; Kraemer et al., 2019 (*26*,*27*)

*CIESIN, Connectivity Lab and Center for International Earth Science Information Network; IBM, individual-based model; MAP, Malaria Atlas Project; UNICEF, The United Nations Children's Fund.

Appendix Table 3. SUDV Transmission Parameters and Data Sources*

Model input	Values/distribution	Reference
Probability of effective transmission per close contact	0.45–0.55 Uniform distribution	Estimated from Xia et al., 2015; Rivers et al., 2014 (*29*,*30*)
Incubation period (days to symptom onset)	Mean: 12 d SD: 4.3 d Lognormal distribution	Estimated from Xia et al., 2015; Rivers et al., 2014 ; Eichner et al., 2011 (*29*,*30*,*34*)
Infectious period	2–21 d Uniform distribution	Xia et al., 2015; Rivers et al., 2014 ; Malvy et al. 2019 (*29*,*30*,*35*)
Percentage of asymptomatic cases	20%	Estimated from Richardson et al. 2016; Mbala et al., 2017 (*32*,*33*)
Time from symptom onset to hospitalization	5 d	Conrad et al., 2016 (*36*)
Days in hospital	10–15 d Uniform distribution	Xia et al., 2015 (*29*)
Case fatality rate by age	39%	CDC 2022 (*31*)
Time from symptom onset to death	5–10 d Uniform distribution	Legrand et al., 2007; Agua-Agum et al., 2015; Robert et al. 2019 (*37*–*39*)
Days infectious before burial after death	2–7 d Uniform distribution	Estimated from Conrad et al., 2016; Potluri et al. 2020 (*36*,*40*)

*SD, standard deviation; WHO, World Health Organization.

Appendix Table 4. Parameters of Ebola nonpharmaceutical interventions*

Model input	Values/ distribution	Reference
Reduction in transmission probability due to use of PPE at health facilities during the postintervention period	70%	Estimated from Dunn et al., 2016; Jefferson et al., 2008 (*44*,*45*)
Probability of seeking treatment at a medical facility in the preintervention period	0.6	Estimated from Conrad et al., 2016 (*36*)
Probability of seeking treatment at a medical facility in the postintervention period	0.8	Estimated from Conrad et al., 2016 and Jalloh et al., 2020 (*36*,*46*)
Probability of hospitalization among individuals seeking treatment in the preintervention period	0.60	Chowell and Nishiura, 2015 (*47*)
Probability of hospitalization among individuals seeking treatment in the postintervention period	0.90	Chowell and Nishiura, 2015 (*47*)
Among deaths in the hospital, probability of having a safe burial	1.0	Assumption based on WHO reports
Among deaths at home, probability of having a safe burial in the preintervention period	0.1	Conrad et al., 2016 (*36*)
Among deaths at home, probability of having a safe burial in the postintervention period	0.95	Conrad et al., 2016 (*36*)
Percentage of contacts (first ring) of infected individuals located by contact tracing team	Beta distribution with mean 70%	Estimated from WHO, 2019b; WHO, 2020 (*42*,*43*)

*FWs, frontline workers; GP, general population; HCWs, healthcare workers; PPE, personal protection equipment; WHO, World Health Organization.

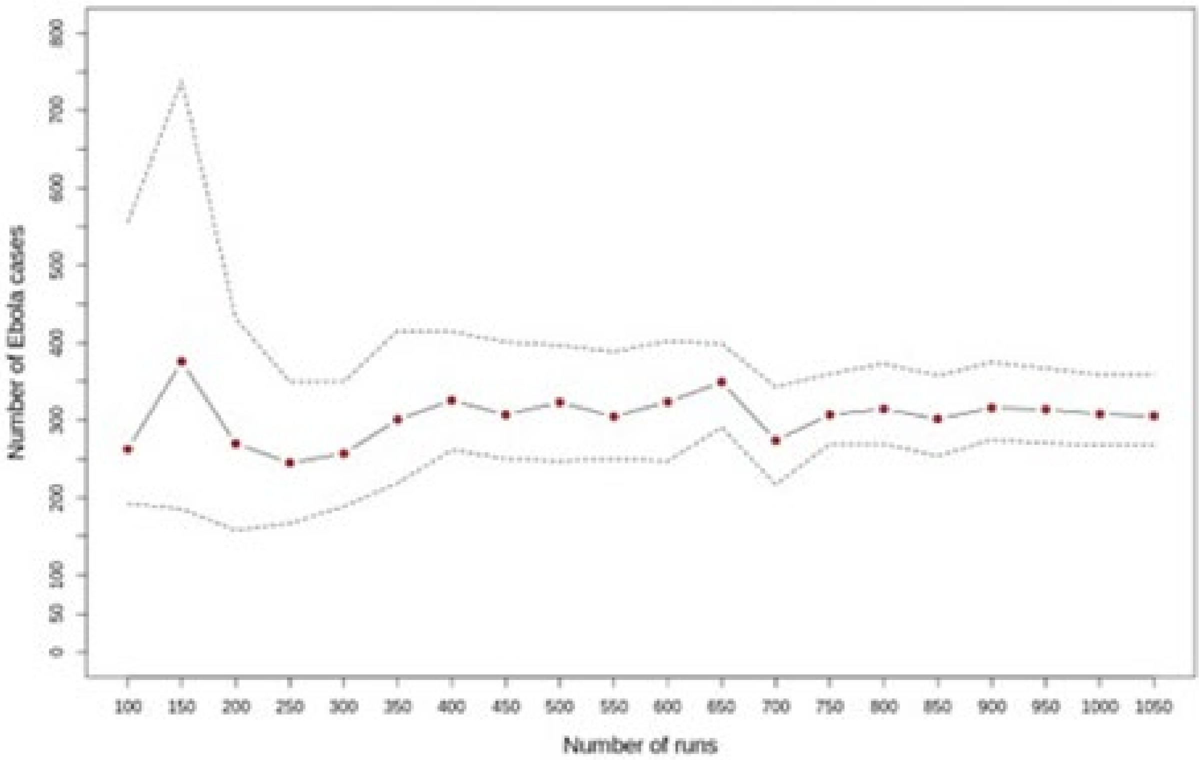

Appendix Figure. Optimal number of runs to perform IBM-SUDV simulations.
